# PANTHER version 14: more genomes, a new PANTHER GO-slim and improvements in enrichment analysis tools

**DOI:** 10.1093/nar/gky1038

**Published:** 2018-11-08

**Authors:** Huaiyu Mi, Anushya Muruganujan, Dustin Ebert, Xiaosong Huang, Paul D Thomas

**Affiliations:** 1 Division of Bioinformatics, Department of Preventive Medicine, Keck School of Medicine of USC, University of Southern California, Los Angeles, CA 90033, USA; 2 School of Life Sciences, Guangzhou University, Guangzhou 510006, China

## Abstract

PANTHER (Protein Analysis Through Evolutionary Relationships, http://pantherdb.org) is a resource for the evolutionary and functional classification of genes from organisms across the tree of life. We report the improvements we have made to the resource during the past two years. For evolutionary classifications, we have added more prokaryotic and plant genomes to the phylogenetic gene trees, expanding the representation of gene evolution in these lineages. We have refined many protein family boundaries, and have aligned PANTHER with the MEROPS resource for protease and protease inhibitor families. For functional classifications, we have developed an entirely new PANTHER GO-slim, containing over four times as many Gene Ontology terms as our previous GO-slim, as well as curated associations of genes to these terms. Lastly, we have made substantial improvements to the enrichment analysis tools available on the PANTHER website: users can now analyze over 900 different genomes, using updated statistical tests with false discovery rate corrections for multiple testing. The overrepresentation test is also available as a web service, for easy addition to third-party sites.

## INTRODUCTION

PANTHER is a comprehensive resource for classification of genes according to their evolutionary history, and their functions (1,2). While the evolutionary and functional classifications in PANTHER are highly correlated, they are not identical. The correlation becomes greater as the evolutionary relationships become closer. The PANTHER evolutionary classification has three levels, from least to most specific: protein class, family, and subfamily. *Protein class* includes both homologous groups (‘superfamilies’ such as protein kinase, comprising multiple divergent families), and groups that are mostly analogous (such as ‘transporter’) but may also include homologs that are too diverged in sequence to reliably establish homology. Each protein class is usually named according to the most common function observed in a family, but it may include members with different functions that share an evolutionary history. A PANTHER *protein family* contains genes that are related to each other by descent from a common ancestor, as established by statistical sequence similarity, and whose sequences can be aligned reliably into a multiple sequence alignment. For each of the over 15 000 families in PANTHER, the detailed relationships between family members are represented in terms of a phylogenetic tree that shows how the family evolved by the processes of speciation, gene duplication, and horizontal transfer (3). Each internal branch point (node) in a phylogenetic tree is labeled according to the type of evolutionary process that caused members of the family to diverge. This family tree is reconstructed from the protein sequences of family members, using a computational inference pipeline that has been described in detail (4,5). Each family tree is further subdivided into *protein subfamilies*. Because of the importance of gene duplication in creating functional diversity within a family (6,7), PANTHER defines subfamilies in terms of gene duplication events. Whenever a gene duplication occurs (except a recent duplication that results in additional genes in only one reference species in PANTHER) a new subfamily is created for the duplicate with the more highly diverged protein sequence. Thus, genes in the same subfamily are very likely to have shared functions based on their common descent, with little divergence, even though these genes are in different species. Pairwise orthologs (pairs of genes that can be traced to the same gene in their common ancestral genome) are also determined directly from the PANTHER trees. PANTHER includes tools for analyzing sequences according to the evolutionary classifications (8,9). Users can browse one or more selected genomes by protein class, using the PANTHER Prowler tool. They can analyze the phylogenetic trees, and the underlying sequence data (in the form of the multiple sequence alignment used to infer the tree) with the PANTHER Tree Viewer tool. Users can upload a new protein sequence to the website, where it is compared statistically (using HMMER3 software (10)) to the ∼80 000 subfamilies that are represented as hidden Markov models (HMMs) (11), to classify it by subfamily (or family, if it does not match a subfamily closely enough).

For functional classification, PANTHER utilizes the Gene Ontology (GO) (12,13). PANTHER employs the Gene Ontology in two different ways, and it is important for users to understand the differences between them. First, PANTHER includes all annotations provided by the Gene Ontology Consortium (available at http://geneontology.org) to the *full Gene Ontology* (comprising ∼45,000 distinct function terms). These annotation sets include all GO evidence codes, and are labeled ‘GO complete’. Second, PANTHER includes inferred annotations to a reduced (‘slim’) classification that includes only a *subset of the Gene Ontology* (comprising 655 distinct function terms in PANTHER versions 9.0 through 13.1, but substantially expanded in PANTHER 14.0 as described below). These annotation sets are labeled ‘PANTHER GO-slim.’ These inferred annotations are produced through annotation of the PANTHER family trees, so they can be directly related to the evolutionary classification. The annotation of the PANTHER family trees is performed by manual curation, in a process that has been described previously (14). Briefly, curators review all experimental GO annotations for all genes in a family, in the context of the phylogenetic tree. They then choose the most informative GO terms to infer gain or loss of each function (GO term) over ancestral branches in the tree. Ancestral functions are then propagated to descendant sequences, except in lineages in which they are annotated with a function loss. This process has a distinct evidence code in GO, IBA, or inferred from biological aspect of ancestor. As a result, the PANTHER GO-slim annotations represent only the subset of GO annotations that have been *selected by curation* (from available experimental annotations), and *judged to be evolutionarily conserved*. Using this process, over 5000 of the PANTHER trees have been annotated to date. It is important to note that while a given gene will belong to only one evolutionary class, it can have many distinct GO terms that describe different aspects of its function. It may also have no known or inferred function (‘function unclassified’). Like the evolutionary classification, GO functions can be of varying specificity, from general terms (e.g. kinase activity) to more specific terms (e.g. tyrosine protein kinase activity). Unlike the evolutionary classification, however, a given GO term will often have more than one parent term, reflecting multiple ‘axes’ of classification. Function classifications and evolutionary classifications generally have a complex relationship. In general, a given Gene Ontology class can contain genes from different evolutionary groups (like subfamilies), but most members of a given subfamily will be associated with the same, or similar, inferred GO terms. In addition to the GO, functional classification in PANTHER also includes biological pathways, both PANTHER pathways (15) and Reactome pathways (16). PANTHER supports several tools for analyzing genes by functional classifications, which have been described in detail (9). Users can upload a list of genes from the PANTHER home page, and retrieve the functional classifications, visualize functional classes as an interactive bar or pie chart, or perform enrichment analysis to find functions that are statistically over- (or under-) represented in a given gene list.

Here, we describe the latest major improvements to the PANTHER resource. The improvements are in two areas: the PANTHER core data, and the PANTHER gene list analysis tools. In the core data, we have increased the number of prokaryotic and plant genomes in the families and phylogenetic trees, and refined hundreds of protein family boundaries. We have created a completely new PANTHER GO-slim and associated annotations, and improved the PANTHER Protein Class for protease and protease inhibitor families. In the gene list analysis tools, we have developed additional software to enable users to easily analyze lists from over 900 genomes (up from 104 in our last update). We have also implemented an additional statistical test method (Fisher's exact test) for the PANTHER overrepresentation test, as well as the Benjamini-Hochberg False Discovery Rate for multiple test correction for all statistical tests on the PANTHER site. The overrepresentation testing tool is also available via web services, so it can be easily added to any third-party website.

## PANTHER CORE DATA IMPROVEMENTS

### More plant, animal and prokaryotic genomes in phylogenetic trees

**Figure 1.**
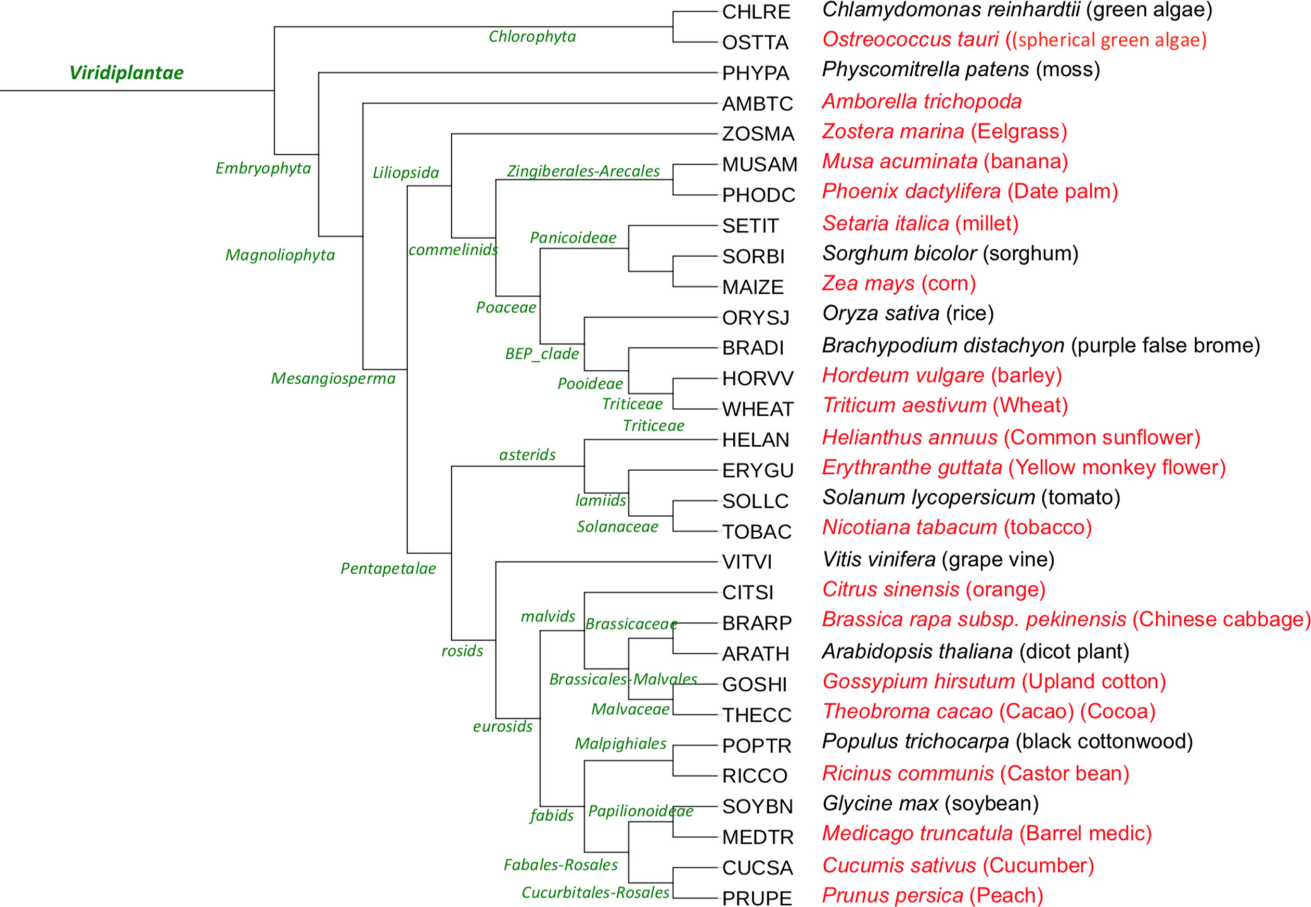
Species tree of plant genomes in PANTHER version 14. New plant genomes are in red.

Since our last update paper, we have added 28 genomes to the reference phylogenetic trees in PANTHER, an almost 30% increase. The new genomes were added in collaboration with two other projects: the Quest for Orthologs (QfO) Consortium (17), and the Phylogenes project (http://www.phylogenes.org/). The new genomes from the QfO collaboration (Table 1) were added primarily to improve sampling of the tree of life. Three bacteria were added and one archaeon, though of course overall sampling of prokaryotes remains low in PANTHER compared to the eukaryotes. In the animals, we added leech as a basal protostome, the red flour beetle as an outgroup insect to the existing fly genomes, and gar as a basal ray-finned fish that diverged prior to the teleost-specific whole genome duplication. In collaboration with the Phylogenes project, we have tripled the number of plant genomes in PANTHER (Figure 1). Most of these are agricultural plants, but one is a basal flowering plant (*Arborella*) and another a single-celled plant (*Ostreococcus*). Users should be aware that many of these plant genomes are polyploid, which can appear in the PANTHER trees as very recent gene duplication events.

**Table 1.** New non-plant genomes since PANTHER 11.0 (2016)

prokaryotes	*Mycoplasma genitalium* (urethritis bacterium)
	*Helicobacter pylori* (stomach ulcer bacterium)
	*Neisseria meningitidis serogroup b* (meningococcus bacterium)
	*Nitrosopumilus maritimus* (marine archaeon)
protostomes	*Helobdella robusta* (leech)
	*Tribolium castaneum* (red flour beetle)
vertebrates	*Lepisosteus oculatus* (spotted gar)
	*Oryzias latipes* (Japanese rice fish)

### Improved family boundaries

The PANTHER team has been collaborating with the Ensembl Compara/TreeFam (18) team on refining family boundaries for inferring phylogenetic trees. The goal was to identify families with low quality multiple sequence alignments, as phylogenetic tree inference depends crucially on these alignments. Low quality alignments generally result from families that are highly diverse in sequence and/or domain structure, and these families would then be reclustered into smaller, more closely related, sequence families. The Ensembl team identified PANTHER families that, when used for collecting homologs from Ensembl gene predictions and aligning them, result in potentially low quality alignments. Specifically, families were identified for which the final, trimmed Ensembl alignments either (i) contain a large proportion (>50%) of family members that do not align to the retained ‘core’ alignment, or (ii) the core alignment is short (<100 columns) and the total alignment length is at least 4 times larger than the core. This process identified 228 families with poor alignments. The PANTHER team used two other criteria to identify additional, overly diverse families. We identified families for which at least 10% of the members each align to less than 30 columns (amino acid sites) of the multiple alignment (98 families), and cases where the family tree contained two or more distinct subtrees where the inter-subtree alignment shared less than 30 amino acid sites in common (549 families). The latter criterion indicates that two or more distinct (essentially non-overlapping) families were incorrectly merged together into a single family. Combining the families identified by each of these criteria resulted in a set of 828 families (less than the sum since a given family might be identified by more than one criterion). These diverse families were subsequently reclustered using the standard PANTHER pipeline as described in (4,5), into 3026 new families in PANTHER 14. To minimize disruption for end users, for each of the original 828 families, the previous PANTHER family identifier has been forward-tracked to the new family with the largest number of former members. All other new families have been given new family identifiers.

### New PANTHER GO-slim, and annotations

Starting in 1998, the PANTHER team independently developed a classification of gene function (PANTHER/X) that included both molecular-level, and pathway-level classes (1). In 2005, we modified the molecular-level classes to become the PANTHER Protein Class ontology, and converted our functional classifications to Gene Ontology (GO) terms (8). Since we used only a small, selected subset of GO terms, we called these function ontologies ‘PANTHER GO-slims,’ one for each of the three aspects of GO: molecular function, biological process, and (from 2007) cellular component. The PANTHER GO-slims have been revised several times since then, but these changes have been relatively minor. Annotations of PANTHER HMMs to the GO-slims, on the other hand, have been updated regularly and extensively.

Over the past two years, we have made a *complete revision* to both the PANTHER GO-slims themselves, and the annotations of genes to these ontologies. Starting in 2017, all legacy PANTHER GO-slim annotations were replaced by the phylogenetic annotations provided by the GO Phylogenetic Annotation project (14). In this project, an expert biocurator reviews all experimentally-supported GO annotations that have been made to all members of a protein family, in the context of the PANTHER phylogenetic tree. The biocurator then chooses the most informative GO annotations and determines (based on other GO annotations as well as properties of the sequences, organisms and evolutionary events such as gene duplication) the ancestral branch in the evolutionary tree that a given GO term (function) was gained (and potentially subsequently lost). This allows prediction of function for sequences with no experimental GO annotations, by propagating function from ancestors to descendants. As of October 2018, over 5500 families have been manually curated, using 8759 distinct GO terms (Table 2). However, until now, these terms have been mapped to the higher-level terms in the older PANTHER GO-slim ontologies, which contained less than 700 terms (<2% of all GO terms).

**Table 2.** Number and frequency of GO terms used in GO Phylogenetic Annotation as of October 2018

# of distinct tree branches annotated with a given GO term	Total number of different GO terms	Cellular component terms	Molecular function terms	Biological process terms
1	4741	443	1648	2650
2–4	2851	427	897	1527
5–10	822	176	197	449
11–50	314	105	93	116
51–100	18	9	6	3
>100	13	11	1	1
Total	8759	1171	2842	4746

For PANTHER version 14, we have dramatically expanded the PANTHER GO-slims, specifically in order to more accurately represent this set of 8759 GO terms used during the manual GO Phylogenetic Annotation process. As described above, these terms were selected by expert review on a family by family basis, from a much larger set of available GO terms, because they were judged to be both informative of function, and evolutionarily conserved. To construct a new PANTHER GO-slim from these terms, we first selected only the terms that were used multiple times. Specifically, we required that a term was used to annotate more than five different tree branches (note that we used the ontology relations to count not only annotations directly to a GO term, but to its more specific descendant terms, i.e. following 'is_a' and 'part_of' relations in the GO graph). We then added in any GO terms that were the common ancestors (in the complete GO graph) of two or more GO terms obtained from the first step, ensuring that all terms could be traced via relations to the root of the ontology. The new PANTHER GO-slim contains 3040 terms, with 2005 biological process, 523 molecular function and 512 cellular component terms. The ontology can be downloaded from http://data.pantherdb.org/PANTHER14.0/ontology/panther_slim.obo. This construction process is fully automatic, and can be regularly updated as the GO Phylogenetic Annotation project proceeds.

### Alignment with MEROPS for proteases

**Figure 2.**
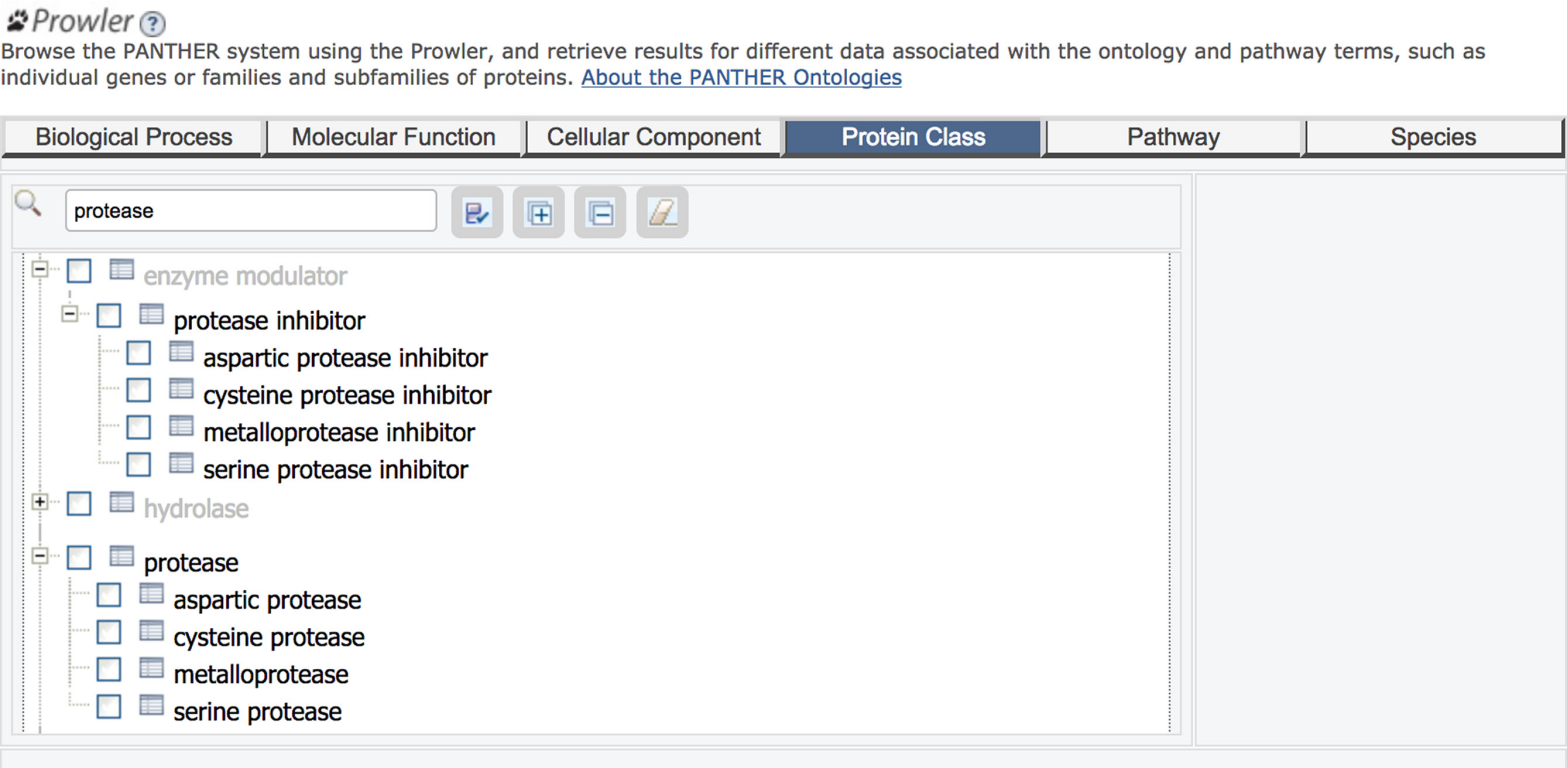
The updated PANTHER Protein Classes *protease* and *protease inhibitor* viewed in PANTHER Prowler. The major subclasses of proteases, such as *aspartic protease* and *metalloprotease*, have been aligned with MEROPS, while families in smaller subclasses (e.g. threonine proteases) or proteases of unknown mechanism, remain directly under the upper level classes.

PANTHER aims to provide a comprehensive classification of protein-coding gene families. We recognize that there are many targeted, family- or function- specific resources on the web, which have been carefully curated, and which could potentially be disseminated through PANTHER. As a first example, published last year (19), we have worked with the MEROPS database of peptidases (proteases) and peptidase inhibitors. In order to align with MEROPS, we modified the PANTHER Protein Class hierarchy for proteases (Figure 2) to match the upper-level classes in MEROPS. We then worked with the MEROPS team to make sure all PANTHER protease families were mapped to families in MEROPS, and assigned to the correct upper-level classes. PANTHER now includes nearly all non-viral protease families in MEROPS. We encourage developers of other family- or function-specific databases to contact us, if they are interested in incorporating their classification information in PANTHER.

## PANTHER GENE LIST ANALYSIS TOOL IMPROVEMENTS

### Analyzing over 800 additional genomes that are not in the PANTHER trees

**Figure 3.**
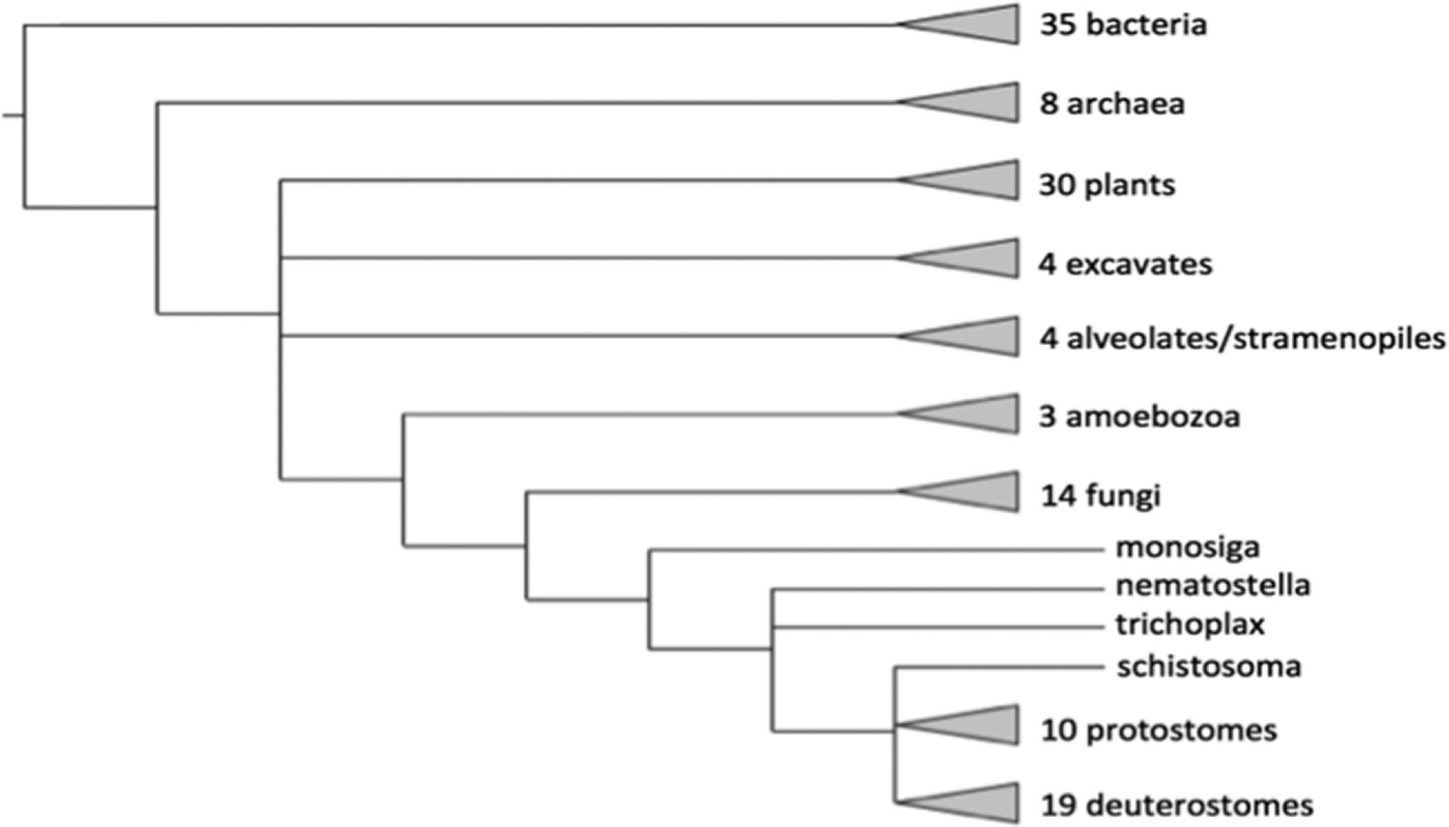
Phylogenetic distribution of genomes available in PANTHER 14.0.

The PANTHER family phylogenetic trees are constructed from 131 genomes (Figure 3), and previously, the PANTHER analysis tools on the website could only be applied to the genomes in the phylogenetic trees. As whole genome sequencing and genome-wide experimentation continue to advance, a growing number of users are working on a wide variety of other genomes. For analysis of other genomes, we have long provided downloadable software for preparing files that could be uploaded to PANTHER for analysis. But many users found the downloadable software difficult to use, particularly if they had limited computational expertise. As a result, one of the most common user requests we have received, is support for additional genomes outside of those in the PANTHER trees.

**Figure 4.**
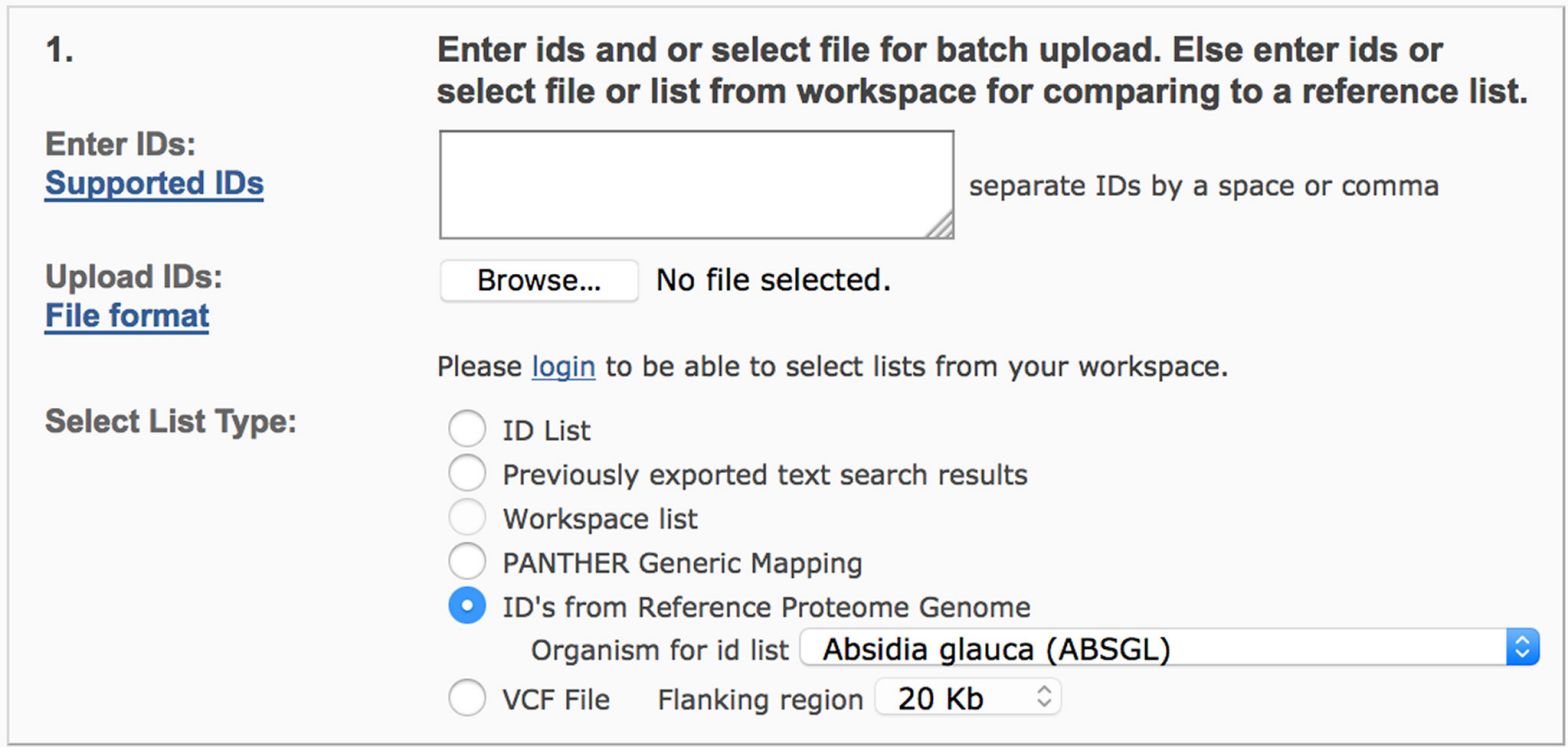
User interface to support an additional 800+ genomes in the PANTHER gene list analysis tools. Users can convert their gene list to a UniProt ID list, upload it and select the list type as ‘ID’s from Reference Proteome Genome’. Then select the organism in the drop-down menu.

To address this problem, in collaboration with InterPro (20) and UniProt Reference Proteomes (21), we have implemented a solution to support over 800 additional genomes on the PANTHER website. Currently we include all UniProt Reference Proteomes with more than 4000 protein-coding genes. We have scored the genes (with UniProtKB identifiers) in these genomes against the PANTHER HMMs in advance, and stored the classification results in the PANTHER database. Users just need to convert their gene list to UniProtKB identifiers, and it can then be analyzed seamlessly on the PANTHER website (Figure 4).

### New defaults with Fisher's exact test, and an FDR correction for multiple testing

Starting in 2004, the PANTHER website has hosted two interactive tools for finding classes of genes that are enriched among the genes in a user's input gene list, relative to a ‘reference’ gene list (from which the input list was selected) (8). The first tool, called the ‘overrepresentation test’, takes the input list (and a ‘reference’ list), and performs a statistical test for over- and underrepresentation: is a given (functional) class found statistically more (or less) often in the input list, than expected by chance? The second tool, called the ‘enrichment test,’ takes the list of all genes that were assayed in an experiment, together with a numerical value (e.g. fold change in expression level) and performs a statistical ‘gene set enrichment’ test: for each (functional) class, the input values are compared to the distribution for all genes, using the Mann–Whitney U Test. In previous versions of PANTHER, for both the overrepresentation test and the enrichment test, *P*-values were adjusted by default using the Bonferroni correction for multiple testing.

**Figure 5.**
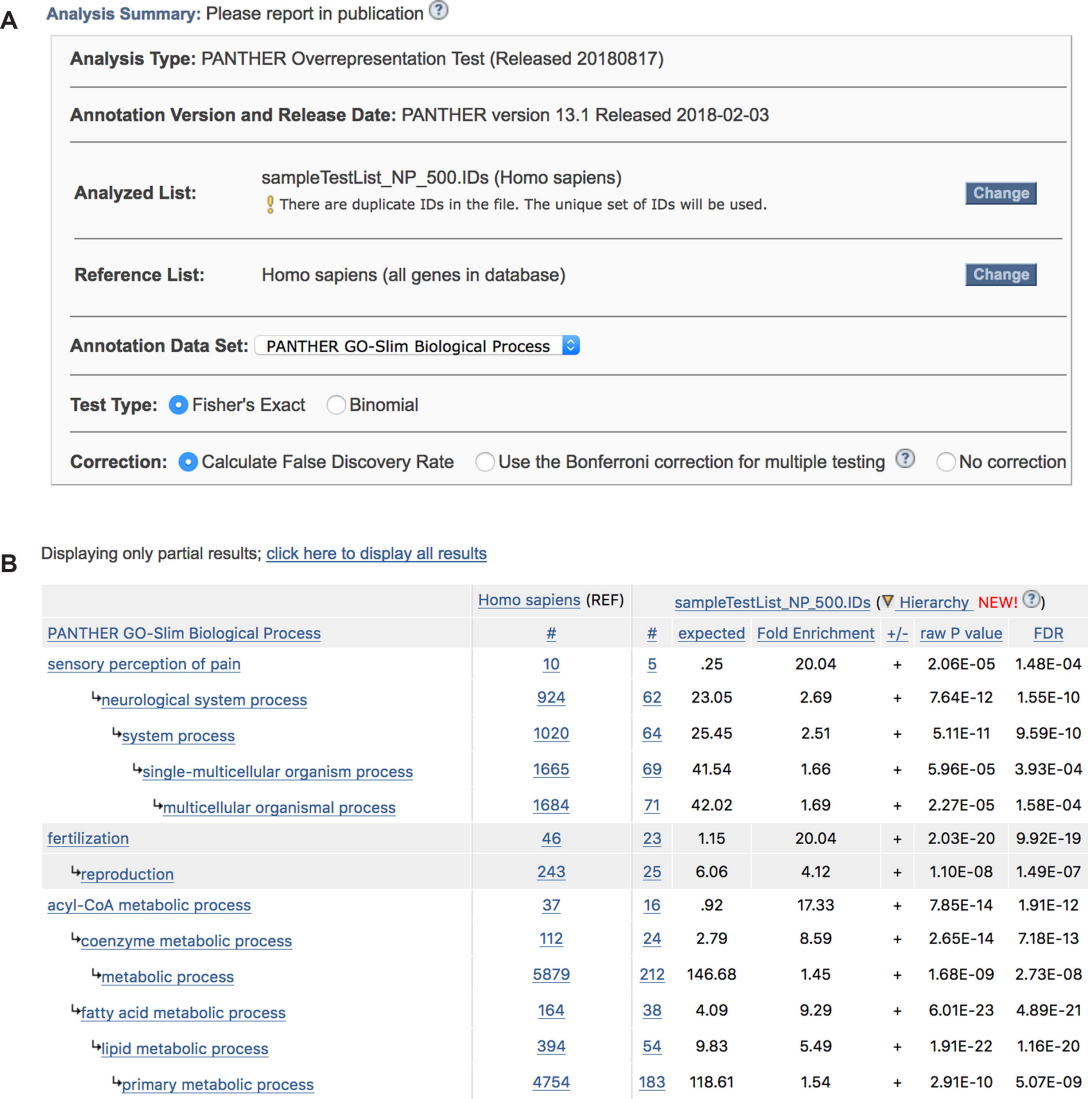
PANTHER overrepresentation test results. (**A**) Analysis options. Users can choose between two different statistical tests, Fisher's Exact and Binomial, and correction methods. (**B**) A screenshot of a result page using the Fisher's Exact test and FDR correction method. Both the raw *P*-values and FDR corrected values are reported in the last two columns.

In the past two years, these tests have been updated in the following ways (Figure 5). First, the overrepresentation test now uses Fisher's exact test by default, rather than the binomial test (i.e. the tool now assumes a hypergeometric distribution by default, which is more accurate for smaller gene lists). Second, both the overrepresentation test and the enrichment test now use the Benjamini-Hochberg False Discovery Rate (FDR) correction by default. The Bonferroni correction was designed for multiple independent tests, and because there are many class-subclass relations in the ontologies used by PANTHER, this correction is too conservative. As a result, using the Bonferroni correction may mask biologically significant results. The FDR was designed to control the false positive rate in the statistical test results, and is generally considered a better choice in enrichment analysis (also called ‘pathway analysis’). The Bonferroni correction is still available as a selection option (Figure 5A), if users need to replicate a previously obtained result, or simply for comparison with the FDR correction.

### How to add the PANTHER overrepresentation tool to a third-party website

The PANTHER overrepresentation testing tool is also available via an Application Programming Interface (API) access. Software developers can use the API to easily integrate the tool into their own (third-party) website. Users can enter a gene list on the third-party site, which can then be sent automatically to the PANTHER overrepresentation tool via the API. The overrepresentation API has two options for returning the statistical test results: either as XML that can be formatted on the third-party site, or as a redirect to the PANTHER site, where the results can be viewed and analyzed using all the tools already available at PANTHER.

Over the past two years, we have added new options to the PANTHER overrepresentation API to provide additional functionality for third-party sites. The API now uses Fisher's exact test with FDR correction by default, with the binomial test and Bonferroni available as options. Crucially, the API now supports a specified reference gene list, in addition to the gene list to be analyzed. Full instructions for the available parameters, as well as example code, are available at http://pantherdb.org/help/PANTHERhelp.jsp#V.E.
